# A Descriptive Profile of Abused Female Sex Workers in India

**DOI:** 10.3329/jhpn.v28i3.5546

**Published:** 2010-06

**Authors:** Subadra Panchanadeswaran, Sethulakshmi C. Johnson, Sudha Sivaram, A.K. Srikrishnan, Carla Zelaya, Suniti Solomon, Vivian F. Go, David Celentano

**Affiliations:** ^1^ Adelphi University School of Social Work, 1 South Avenue, Garden City, New York, NY 11530, USA; ^2^ YRG Centre for AIDS Research and Education, Voluntary Health Services Taramani, Chennai 600 113, India; ^3^ Bloomberg School of Public Health, Johns Hopkins University, 615 North Wolfe St., Baltimore, MD 21205, USA

**Keywords:** Descriptive studies, HIV, Spouse abuse, Violence, Violence against women, India

## Abstract

This descriptive study presents the profiles of abused female sex workers (FSWs) in Chennai, India. Of 100 abused FSWs surveyed using a structured questionnaire, severe forms of violence by intimate partners were reported by most (98%) respondents. Of the total sample, 76% experienced violence by clients. Sexual coercion experiences of the FSWs included verbal threats (77%) and physical force (87%) by intimate partners and forced unwanted sexual acts (73%) by clients. While 39% of the women consumed alcohol before meeting a client, 26% reported that their drunkenness was a trigger for violence by clients. The findings suggest that there is an urgent need to integrate services, along with public-health interventions among FSWs to protect them from violence. Recognition of multiple identities of women in the contexts of intimate relationships versus sex work is vital in helping women to stay safe from adverse effects on health.

## INTRODUCTION

Violence against women has emerged as a pervasive social problem and a public-health issue. Furthermore, violence is often perpetrated by intimate/cohabiting partners of women (1, 2). Recent data indicate that the prevalence of physical and/or sexual intimate partner violence ranges from 15% to 71% globally (3). In the Indian context, patriarchal cultural norms and traditionally-prescribed gender roles intensify the vulnerability of women to violence (4–6). Recent data from the National Family Health Survey (NFHS 3) in India showed that 35.5% of married Indian women experienced physical violence with or without sexual violence from their husbands (7). Research has also documented the multiple forms of abuse reported by women in India, including psychological, verbal, physical, sexual, and economic (8–10). Abuse of wife in India has been associated with insufficient dowries (9, 11), lower levels of male education and poverty (12, 13), premarital and extramarital sex (14), assertive behaviours of respondents (9), and men witnessing violence between parents (15). Violence against women impacts the health of women adversely, with consequences ranging from injuries, reproductive health problems, chronic and debilitating illnesses, to emotional distress and mental health problems (1, 16–18), and, most recently, HIV/AIDS (7, 19–23).

Violence against female sex workers (FSWs) has received inadequate attention from researchers and practitioners alike. Only recently, studies around the world have highlighted FSWs’ experiences of harassment, physical violence, and rape in the context of HIV/AIDS research (24–32). Studies on FSWs in India have highlighted their multiple vulnerabilities stemming from independent solicitation of clients of street-based sex workers that places them at higher risks of violence, rape, and exploitation (33, 34), inability to negotiate safe sex, and risk for sexually transmitted infections (STIs) and HIV (33–38), especially in the context of alcohol-use by males (39–41).

Much of the research on sex work in India and around the world has focused on HIV prevention, condom-use experiences, and interactions of FSWs with clients. There is little research that describes intimate relationships of FSWs, experiences of violence by intimate partners, and sexual coercion. FSWs have often been excluded from the mainstream discourses on women's experiences of violence in intimate relationships, possibly due to the assumption of higher risk for violence and adverse consequences from clients compared to intimate partners (42). Only recently, research evidence has underscored the many dimensions of intimate relationships of FSWs in India, ranging from relationships with ‘regular’ clients who provide emotional succour and financial support (42) to those who perpetrate physical and sexual violence (43). Also, while there is a significant body of research on experiences of intimate partner violence (IPV) among women in the general population, documentation of these experiences among FSWs is scanty. It is unclear if experiences of FSWs are similar to or different from women in the general population. Further, given the context of criminalization and stigmatization of sex work in India, FSWs who experience violence from partners or clients are unwilling to disclose or report the same to police or seek help (44). Some researchers have recently stressed the importance of recognizing multiple identities of FSWs (45), understanding individual contexts of women's lives in sex work (46), paying heed to FSWs’ risk for HIV infection from not only clients but also non-commercial intimate partners (37, 47–50). A recent study of ‘Devadasis’ (traditional form of sex work) in India underscored the significance of intimate relationships of FSWs with ‘regular’ clients who provide emotional succour, financial support, and share children (42).

The goal of this exploratory study was to gain an in-depth understanding of street-based female sex workers’ experiences of violence in intimate and work spheres. Further, this study will contribute to existing extant literature on this issue and extend it by providing a holistic view of the contexts of street-based sex workers’ differential experiences of violence from intimate partners/regular non-paying long-term partners, and violence from paying clients, including experiences of sexual coercion in India. Specifically, the objectives of the present study were to: (a) describe and compare FSWs’ experiences of physical violence from clients and intimate partners; (b) outline the triggers for violent episodes, including alcohol-use; and (c) explore FSWs’ experiences of threats and actual occurrences of sexual coercion from clients and intimate partners.

## MATERIALS AND METHODS

### Sample and recruitment

The study was nested within a five-country NIMH Collaborative HIV/STD Prevention Trial that seeks to test the efficacy of HIV-prevention messages delivered through community popular opinion leaders (CPOLs). The CPOLs are influential individuals in local communities, who are approached by friends for advice and counselling (51). In India, the extensive formative research phase revealed two groups most at-risk for HIV infection: female sex workers and men who frequented local wine-shops (52–54). The present study was part of the team's efforts to gain an in-depth understanding of the intersections of violence and HIV risk for FSWs in Chennai.

For the study, trained field staff contacted key-informants and CPOLs from various cruising locations, such as cinema halls, bus-terminals, and hotels/lodges in Chennai during their regular, bi-weekly field-trips and informed them about the study and enlisted their support in recruiting participants. The main method of recruitment was through word of mouth referrals to other women in their social networks. When potential respondents were identified, they were provided contact information of the project staff who subsequently approached the FSWs for participation in the study. Data on ineligibility and refusal rates were not recorded.

The eligibility criteria included: (a) having been a female sex worker for at least one year; (b) being in an intimate relationship with a non-paying male sexual partner currently or in the past year; (c) experiencing at least one form of violence (verbal/physical/sexual) either from a client and/or an intimate partner in the past year; and (d) soliciting clients on streets and public venues, such as cinema halls, bus-terminals, railway stations, hotels/lodges and/or independently through brokers, through informal social networks and providing sexual services at a venue of the client's choice. The field staff determined eligibility in a conversational manner, and while all the required questions were asked of potential respondents, they did not follow any rigid sequence when questioning. The specific questions included: “How long have you been practising sex work?” “Where do you generally solicit clients?” “Do you have someone with whom you feel very close to at this point or have been in the past year, someone who you have sex with, but who does not pay you?” “Is the person male or female?” “In your relationship with this intimate partner, have you experienced any form of violence in the past year?” “Has any paying-client been abusive to you in the past year?” FSWs who did not report being in an intimate relationship in the past year, those who identified their intimate partners as female (however, there were none reported), and those who did not report any experience of violence in the past year were ineligible for the study.

For the purposes of the study, ‘male intimate partner’ of FSWs was defined as: (a) husbands/spouses who cohabited with FSWs, or (b) cohabiting ‘regular, non-paying male partner’ whom women considered ‘husbands’ (possibly ex-client), or (c) non-cohabiting ‘regular, non-paying male partner (possibly ex-client). Clients of FSWs in the study were defined as those ‘paying-sexual partners’ (one-time clients) with whom women did not have an ongoing relationship.

### Measurement

The survey instrument was pilot-tested among 20 FSWs, revised, and subsequently finalized. Besides basic demographic information, the questionnaire included the following:

**Experiences of violence from partners and clients:** A modified version of the multi-country study protocol of the World Health Organization (55), developed for cross-cultural use, was used for measuring the respondents’ experiences of violence. Experiences of verbal and physical violence by the current intimate partner and/or client were assessed in the 12 months before the interview. Verbal abuse was assessed with one item that examined yelling or shouting, and moderate physical violence included slapping/throwing, pushing/pulling, and hitting with fist while severe physical violence included kicking/dragging, trying to burn/strangle, threatening with a knife/gun/other weapons, and attacking with a knife/gun/other weapons. Additionally, the frequency of each of the acts of violence was also assessed. The Cronbach's alpha for the abuse items for this sample was 0.69 for IPV and 0.71 for violence from client.

**Injury:** Women were asked if they experienced sprains/bruises/cuts/scratches/ aches/physical pain, injury/broken bones, had lost consciousness, or had to visit a doctor or a health centre because of violence from intimate partner or client.

**Experiences of sexual coercion:** Experiences of sexual coercion included verbal threats, physical force, and force to perform unwanted sexual acts.

**Alcohol-use:** Alcohol-use in the case of IPV was assessed with a set of four questions. Women were asked if they/their partners were under the influence of alcohol during the most recent episode of violence. They were also asked about the frequency of alcohol consumption by women/partners during episodes of violence in the past year. Further, women were asked if they generally consumed alcohol before meeting a client/regular customer and if clients generally consumed alcohol before sex.

The protocol also included questions on the ‘main reasons’ that intimate partners and/or clients assaulted them for in the past year. The survey instrument was forward-translated into Tamil and back-translated into English.

### Analysis of data

Given the goals of the study, analyses conducted were primarily descriptive and exploratory. Univariate descriptive analysis was performed to examine the distribution of all variables of interest. The Stata software (version 9.0) was used for quantitative analyses. Variables for experience of violence from client and IPV were coded as four-level categorical variable: 0=no violence, 1=verbal violence, 2=moderate physical violence, and 3=severe physical violence. Exploratory analyses, i.e. crude associations and chi-square analyses, were conducted to investigate the potential factors associated with experiencing sexual coercion from clients among the study FSWs.

### Ethics

The study staff completed surveys after ascertaining eligibility and obtaining informed consent during March-July 2004. The protocols and procedures were approved by the institutional review boards of both Johns Hopkins University and YRG Centre for AIDS Research and Education (YRG CARE) in Chennai. In total, 100 sex workers were surveyed using the structured questionnaire.

## RESULTS

### Characteristics of street-based female sex workers in Chennai

The sociodemographic characteristics of street-based FSWs are summarized in Table 1. Their mean age was 32.3 years (SD 5.3), and most (81%) had received education up to either elementary or middle-school levels. For most (81%) respondents, intimate partners comprised husbands or regular non-paying sexual partners who cohabited with the women. Women had been in the sex-trade for, on average, six years (SD 3.6).

**Table 1.** Sociodemographics of abused female sex workers (n=100) in Chennai, India

Demographics	IPV* (n=100)		Violence from client (n=76)	
	No.	%	No.	%
Age				
Average years (mean, SD)	32.3 (5.3)		31.8 (5.5)	
20–29	28	28	25	32.9
30–39	62	62	45	59.2
≥40	10	10	6	7.9
Education				
No schooling	19	19	15	19.7
Elementary schooling	49	49	41	54.0
Mid-level schooling	32	32	20	26.3
Marital status				
Unmarried and living alone	5	5	15	19.7
Currently married and living with spouse	76	76	56	73.4
Unmarried and living with intimate partner	5	5	5	6.6
Deserted	6	6	4	5.3
Widowed	8	8	8	10.5
Years working in sex-trade				
1–4	33	33	30	39.5
5–9	47	47	34	44.7
10–14	14	14	9	11.8
≥15	6	6	3	4.0

*All the female sex workers in the study experienced violence from their intimate partner;

IPV=Intimate partner violence;

SD=Standard deviation

### Triggers for violent episodes and experiences of various forms of violence and injury

Triggers for violence ranged from ‘no particular reason’ to ‘not completing household chores to satisfaction’ (Table 2). The most common trigger reported by the respondents for both intimate and client-related violence was arguments over money. In the case of IPV, this was followed by refusal of women to have sexual relations (83%), partner's suspicion of being unfaithful (81%), and drunkenness of partner (81%). Initiating condom-use and drunkenness of women were important triggers for client-related violence (46% and 26% respectively).

**Table 2.** Type of violence and triggers for violence that FSWs experienced from their intimate partners and clients

Type of violence and triggers	IPV (n=100)		Violence from client (n=76)	
	No.	%	No.	%
Type of abuse				
Verbal aggression	99	99	75	98.7
Minor physical assault	100	100	31	40.8
Severe physical assault	98	98	25	32.9
Individual type of violence				
Yelled or shouted at	99	99	75	98.7
Slapped or had something thrown at them	98	98	14	18.4
Pushed, pulled, or held down	97	97	30	39.5
Hit with fist or something that could hurt them	92	92	4	5.3
Kicked or dragged	97	97	25	32.9
Tried to burn or strangle	62	62	0	0
Threatened with knife, gun, or other weapons	39	39	0	0
Attacted with knife, gun, or other weapons	32	32	0	0
Type of trigger*				
No particular reason	69	69	8	10.5
Husband or client was drunk	81	81	57	75.0
Initiated condom-use	10	10	44	57.9
Suspected of being unfaithful	81	81	49	64.5
Argument over money	100	100	75	98.7
Husband had work tensions	38	38	NA	
Refused sexual relation	83	83	NA	
Retorted back to husband/elders	77	77	NA	
Disobeyed husband/elders	69	69	NA	
Housework not completed to satisfaction	53	53	NA	
Was drunk	NA		26	34.2

*Not all triggers were asked in regard to IPV and violence from client.

NA in the table indicates that the particular trigger was not asked;

FSWs=Female sex workers;

IPV=Intimate partner violence

Experience of violence from either client or intimate partner was a criterion for participation in the study. The results showed that, although 24% of the participants did not report any experience of violence from client, all the respondents experienced some form of IPV in the past year. While all the FSWs experienced some form of violence, significantly more numbers experienced ‘severe IPV’ (98%) compared to that of severe client-initiated violence (Table 2). Specifically, 62% reported that their intimate partners had tried to burn/strangle them in the past year, and none reported these experiences from clients. Further, significant proportions of the women experienced severe physical assaults from intimate partners ‘many’ times in the past year, including kicking/dragging (61.9%), attempt to burn/strangle (45.2%), threats with a knife/gun/weapon (38.5%), and even being attacked with a knife/weapon (15.6%) (data not shown). On the other hand, the most common form of abuse from clients was verbal aggression (98.7%) in the past year (Table 2).

The study respondents suffered significant injuries because of IPV and relatively fewer consequences of violence from client. Sprains/bruises/cuts/scratches/aches/physical pain were the most commonly-reported form of injuries from both clients and intimate partners. However, while 81% of the women reported broken bones because of IPV, only two reported the same froms client-induced violence. Significantly, 38% reported having lost consciousness due to IPV while none reported the consequence as a result of client-induced violence. Seventy-nine percent of the respondents visited a doctor/sought help from a health clinic to address the injuries due to IPV compared to five women who did so due to client-induced violence.

### Role of alcohol in FSWs’ experiences of violence

The role of alcohol in women's intimate and work-life is shown in Table 3. Most (99%) respondents reported that their intimate partners had been under the influence of alcohol in the most recent episode of violence. Over one-third (39%) of the FSWs also reported consuming alcohol before meeting clients. Consumption of alcohol by the women was not statistically associated with their experiences of verbal/moderate physical IPV or severe IPV.

**Table 3.** Alcohol-use patterns of FSWs and intimate partners

Alcohol-use patterns	FSWs (n=100)
Under the influence of alcohol in the most recent episode of violence	
FSW	17
Intimate partner	99
Frequency of alcohol consumption in violent episodes in the past year (FSW)	
Often	3
Sometimes	8
Rarely/never	89
Frequency of alcohol consumption in violent episodes in the past year (partner)	
Often	92
Sometimes	6
Rarely/never	2
Consumption of alcohol by FSWs before meeting clients generally	
No	61
Yes	39

FSWs=Female sex workers

### Experiences of sexual coercion

In the current sample of abused FSWs, one-third experienced verbal threats, 38% reported physical force from clients to have sex, and 73% reported being forced to perform unwanted sexual acts by clients (Fig.). Sex workers who were physically forced by clients to have sex were more likely to also report experiencing verbal/moderate physical violence from clients compared to those who were not forced to have sex (χ^2^=5.40, p=0.02). We further examined the potential demographic characteristics associated with sexual coercion from client (Table 4). The results showed that the age of women and the number of years working in the sex-trade were significantly associated with experiencing sexual coercion from clients. Given the high correlation between the age of women and the number of years working in the sex-trade, these variables were not adjusted in a logistic regression model; instead, crude associations between the variables of interest were examined for exploratory purposes. The results of bivariate analysis showed that women who had been in the sex trade for 1–4 years had almost five times the odds of being sexually coerced by clients compared to FSWs who had been in the sex-trade for 10 or more years [crude odds ratio (OR): 4.8, 95% confidence interval (CI) 1.2–19.1). Similarly, women who were aged 20–29 years had more than three times the odds of being sexually coerced by clients compared to FSWs who were aged 30 years or older (crude OR: 3.7, 95% CI 1.1–13.4).

**Fig.**
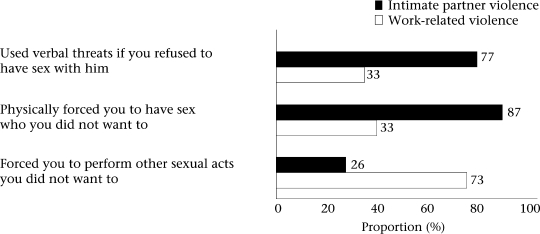
FSWs' experiences of sexual coercion (n=100)

FSWs=Female sex workers

**Table 4.** Association of demographic factors, alcohol consumption, and sexual coercion from clients*

Demographics	Actual experience of sexual coercion				p value
	Yes (n=75)		No (n=25)		
	No.	%	No.	%	
Age (years)					
20–29	25	89.3	3	10.7	
30–39	46	74.2	16	25.8	0.01
≥40	4	40.0	6	60.0	
Education					
No schooling	15	79.0	4	21.0	
Elementary schooling	39	79.6	10	20.4	0.36
Mid-level schooling	21	65.6	11	34.4	
Marital status					
Unmarried and living alone	2	40.0	3	60.0	
Currently married and living with spouse	59	77.6	17	22.4	
Unmarried and living with intimate partner	5	100.0	0	0	0.11
Deserted	3	50.0	3	50.0	
Widowed	6	75.0	2	25.0	
Years working in the sex-trade					
1–4	29	87.9	4	12.1	
5–9	34	72.3	13	27.7	
10–14	12	60.0	8	40.0	0.03
≥15	2	33.3	4	66.7	
Alcohol					
FSWs consume alcohol before meeting clients					
Yes	28	71.8	11	28.2	0.64
No	47	77.0	14	23.0	
Frequency of alcohol consumption from clients					
Always/often	70	77.0	21	23.0	0.22
Sometimes/rarely	5	55.6	4	44.4	

*Sexual coercion was defined as FSW who had actual experiences of sexual coercion from client, not just verbal threats;

FSW=Female sex worker

The large majority of the study women reported that their intimate partners used verbal threats if they refused sex (77%) and had physically forced to have sex (87%) (Fig.). A relatively-smaller proportion (26%) reported that their partners forced them to perform unwanted sexual acts. Further, 86% of the FSWs who were physically forced by their intimate partners to have sex also experienced severe physical IPV, although this relationship was not significant.

## DISCUSSION

The present study captures the FSWs’ differential experiences of abuse in intimate relationships versus violence that emanated from their work-sphere in a purposive convenience sample of abused street-based FSWs in Chennai, India. Experiences of violence from clients among the study women echoed the findings of earlier research on street-based sex workers in India and elsewhere in the world (24, 25, 29, 30, 32, 33), highlighting the dangerous environments in which street-based FSWs operate. Women who were relatively inexperienced in the sex-trade had significantly higher odds of being forced to have sex and perform unwanted sexual acts by clients who exerted more power in the context of illegal sex work in India, as in earlier studies in Australia (56). These problems were exacerbated in the context of alcohol-use by clients, intimate partners, and sometimes women themselves. These findings resonate with those from earlier studies that have underscored substance-use as a means for FSWs to cope with the stressors of sex work (57).

One of the most disturbing findings of the present study was the pervasiveness of reports of severe IPV and injuries unlike previous studies which found that sex work helped women lead autonomous life, independent of abusive and unfaithful partners (46). This finding echoes the previous findings of quantitative and qualitative research in India that found overwhelming reports of severe IPV (9, 10, 48). Interestingly, the present study also found that one of the important triggers for IPV among FSWs was the suspicion of infidelity on the part of women, a finding similar to that found in studies that examined triggers for spousal violence among married women (9, 10, 58). Further, reports of sexual coercion and threats by intimate partners of FSWs were also similar to reports of population-based studies of married women in India (7) and earlier studies with FSWs in India (43). This finding is particularly significant given the established link between sexual coercion of Indian women by their husbands and vulnerability to HIV infection (7). The above findings are especially salient given earlier research evidence that FSWs in Chennai consider their relationship with long-term non-paying, regular partners identical to a matrimonial relationship, and most did not initiate condom-use with their intimate sexual partners (48). It is also possible that the FSWs in our study were unable or unwilling to transcend traditional, patriarchal community gender norms and role expectations in the context of intimate, particularly marital-like relationships with emotional ties with intimate partners as in earlier research among Indonesian sex workers (45), unlike their relationships with paying-clients where they were sometimes able to assert themselves (48).

### Limitations

While this study provides an overview of the abusive experiences of street-based FSWs in India, some limitations of this study need mention. First, the convenience sample of abused FSWs in this study may not be representative of the larger heterogeneous sex worker community in Chennai or elsewhere in India. In addition, the small sample size and selection in this study limited the use of advanced statistical analyses and providing prevalence estimates; these results would need to be replicated in larger cross-sectional and in longitudinal studies. Finally, the findings of the present study have limited generalizability to those FSWs who have experienced violence. Despite these limitations, the study provides important insights into the lives of abused street-based sex workers and highlights their vulnerabilities resulting from violence and experiences of sexual coercion in the contexts of both work and intimate relationships in India.

### Conclusions

In conclusion, it appears that, for abused FSWs, the risk of HIV infection emanates from both their intimate partners and clients. Given the vulnerability of FSWs to violence from client due to condom initiation, it would be important to examine the efficacy of programmes that emphasize condom-promotion efforts initiated by sex workers themselves. Future prevention interventions with the female sex worker community may also need to include specific programmes with male clients. There is an urgent need to recognize that FSWs often must negotiate multiple roles and identities in the contexts of intimate relationships and the challenges that arise in their efforts to stay safe. It is also vital to examine how FSWs cope with their emotional needs and challenges to sexual health in long-term intimate relationships (56).

Understanding the relationship among sex workers’ experiences of violence, alcohol-use (of partners and by women themselves), and HIV risk behaviours is critical for the development of appropriate prevention strategies and policies. Specifically, it would be important to examine if violence itself may be a pathway to sex work and HIV risk behaviours for sex workers in India. Studies in the future would need to not only explore direct and indirect health risks of sex workers stemming from direct exchanges with customers but also move to addressing the same with regular/consistent clients and intimate partners (47, 49, 56, 59). Future research also needs to ensure that female sex workers are included in studies that focus on establishing the prevalence of IPV in the general population and also in interventions that target partner violence along with other women, in addition to programmes that aim at preventing client-initiated violence specific to sex workers. It is imperative that researchers, practitioners, and policy-makers adopt a participatory, holistic approach within the larger context of a human rights-based framework while planning interventions and policies for FSWs (60, 61). Finally, future interventions and policies would need to adopt a multi-pronged approach that addresses structural, contextual, and individual factors that extend beyond the narrow HIV-prevention models keeping overall well-being of sex workers in mind.

## ACKNOWLEDGEMENTS

The National Institute for Mental Health (Grant No. U10 681543-01) provided funds to the last author.

Versions of this article were presented at the International Conference on Violence, Abuse, and Trauma, in San Diego, CA, 2006 and at the International Conference on HIV/AIDS, Toronto, Canada, 2006.

The authors thank Ms Tzu Chang for her help with analysis of data. The authors acknowledge the support of the participating agencies and express gratitude to all the female respondents who freely shared their very personal experiences of violence.
